# Do ^2^H and ^18^O in leaf water reflect environmental drivers differently?

**DOI:** 10.1111/nph.18113

**Published:** 2022-04-12

**Authors:** Lucas A. Cernusak, Adrià Barbeta, Rosemary T. Bush, Rebekka Eichstaedt (Bögelein), Juan Pedro Ferrio, Lawrence B. Flanagan, Arthur Gessler, Paula Martín‐Gómez, Regina T. Hirl, Ansgar Kahmen, Claudia Keitel, Chun‐Ta Lai, Niels C. Munksgaard, Daniel B. Nelson, Jérôme Ogée, John S. Roden, Hans Schnyder, Steven L. Voelker, Lixin Wang, Hilary Stuart‐Williams, Lisa Wingate, Wusheng Yu, Liangju Zhao, Matthias Cuntz

**Affiliations:** ^1^ 8001 College of Science and Engineering James Cook University Cairns Qld 4878 Australia; ^2^ 16724 BEECA Department of Evolutionary Biology, Ecology and Environmental Sciences Universitat de Barcelona Barcelona Catalonia 08028 Spain; ^3^ 3270 Department of Earth and Planetary Sciences Northwestern University Evanston IL 60208 USA; ^4^ Faculty of Regional and Environmental Sciences – Geobotany University of Trier Trier 54296 Germany; ^5^ ARAID‐Departamento de Sistemas Agrícolas Forestales y Medio Ambiente Centro de Investigación y Tecnología Agroalimentaria de Aragón (CITA) Zaragoza 50059 Spain; ^6^ Department of Biological Sciences University of Lethbridge Lethbridge AB T1K 3M4 Canada; ^7^ WSL Swiss Federal Institute for Forest, Snow and Landscape Research Birmensdorf 8903 Switzerland; ^8^ INRAE Bordeaux Sciences Agro UMR ISPA Villenave d'Ornon 33140 France; ^9^ Centre Tecnològic Forestal de Catalunya (CTFC) Solsona Catalonia 25280 Spain; ^10^ Technische Universität München Lehrstuhl für Grünlandlehre Freising‐Weihenstephan 85354 Germany; ^11^ 27209 Department of Environmental Sciences–Botany University of Basel Basel 4056 Switzerland; ^12^ School of Life and Environmental Sciences Sydney Institute of Agriculture The University of Sydney Camden NSW 2006 Australia; ^13^ 7117 Department of Biology San Diego State University San Diego CA 92182 USA; ^14^ Department of Biology Southern Oregon University Ashland OR 97520 USA; ^15^ 3968 College of Forest Resources and Environmental Science Michigan Technological University Houghton MI 49931 USA; ^16^ Department of Earth Sciences Indiana University–Purdue University Indianapolis Indianapolis IND 46202 USA; ^17^ Research School of Biology The Australian National University Canberra ACT 2601 Australia; ^18^ 12381 Key Laboratory of Tibetan Environmental Changes and Land Surface Processes Institute of Tibetan Plateau Research Chinese Academy of Sciences Beijing 100101 China; ^19^ 53045 Shaanxi Key Laboratory of Earth Surface System and Environmental Carrying Capacity College of Urban and Environmental Sciences Northwest University Xi'an 710127 China; ^20^ Université de Lorraine AgroParisTech INRAE UMR Silva Nancy 54000 France

**Keywords:** evaporative enrichment, isotopic biomarker, leaf water, relative humidity, stable isotopes

## Abstract

We compiled hydrogen and oxygen stable isotope compositions (δ^2^H and δ^18^O) of leaf water from multiple biomes to examine variations with environmental drivers. Leaf water δ^2^H was more closely correlated with δ^2^H of xylem water or atmospheric vapour, whereas leaf water δ^18^O was more closely correlated with air relative humidity. This resulted from the larger proportional range for δ^2^H of meteoric waters relative to the extent of leaf water evaporative enrichment compared with δ^18^O. We next expressed leaf water as isotopic enrichment above xylem water (Δ^2^H and Δ^18^O) to remove the impact of xylem water isotopic variation. For Δ^2^H, leaf water still correlated with atmospheric vapour, whereas Δ^18^O showed no such correlation. This was explained by covariance between air relative humidity and the Δ^18^O of atmospheric vapour. This is consistent with a previously observed diurnal correlation between air relative humidity and the deuterium excess of atmospheric vapour across a range of ecosystems. We conclude that ^2^H and ^18^O in leaf water do indeed reflect the balance of environmental drivers differently; our results have implications for understanding isotopic effects associated with water cycling in terrestrial ecosystems and for inferring environmental change from isotopic biomarkers that act as proxies for leaf water.

## Introduction

The stable isotope composition of hydrogen and oxygen in leaf water varies throughout the day, among plants within a site and across environmental gradients (Zundel *et al*., 1978; Flanagan *et al*., 1991a; Cernusak *et al*., 2002, 2016; Lai *et al*., 2008; West *et al*., 2008). Leaf water becomes enriched in the heavy isotopes ^2^H and ^18^O compared with the water entering the roots as a result of evaporative isotopic fractionation during transpiration (Gonfiantini *et al*., 1965). There is also isotopic exchange between water vapour in the atmosphere and that in the leaf (Craig & Gordon, 1965); notably, this continues even if transpiration has ceased under a saturated atmosphere (Welp *et al*., 2008; Kim & Lee, 2011; Helliker, 2014; Goldsmith *et al*., 2017). Furthermore, the distribution of isotope enrichment within the leaf can vary as a function of leaf anatomy and physiology (Yakir *et al*., 1989; Gan *et al*., 2002; Holloway‐Phillips *et al*., 2016; Barbour *et al*., 2021). Therefore, the stable isotope composition of leaf water provides an information‐rich isotopic signal that can be applied across a broad range of disciplines (Yakir, 1998). Interest in understanding leaf water stable isotope composition has been further motivated by recognition that leaf water is the starting point for isotope signals in plant organic compounds such as sucrose, starch, cellulose, lignin, leaf waxes (Yakir, 1992; Farquhar *et al*., 1998; Barbour, 2007; Lehmann *et al*., 2020). Leaf water isotopic signals can even be reflected in the bones and teeth of herbivores, such as kangaroos (Ayliffe & Chivas, 1990; Faith, 2018).

Models of leaf water stable isotope composition have been developed over several decades and typically perform reasonably well at explaining observed leaf water isotopic variation (Dongmann *et al*., 1974; Flanagan *et al*., 1991b; Roden & Ehleringer, 1999; Farquhar & Cernusak, 2005; Cuntz *et al*., 2007; Ogée *et al*., 2007). However, some questions about subtler aspects of leaf water isotopic composition remain (Cernusak *et al*., 2016). One such question is whether stable isotopes of hydrogen and oxygen reflect differently the balance of environmental and physiological drivers that lead to variation in leaf water stable isotopes.

Models of leaf water isotopic composition do not differentiate between hydrogen and oxygen in their general formulation; the major mechanisms that cause leaf water to change isotopically are common to both elements. However, the magnitudes of the fractionation factors associated with the mechanisms do differ. This is also true for meteoric waters, such that the relative extent of variation in the isotopic composition of plant source water and atmospheric vapour across the landscape is different between ^2^H and ^18^O; on average, there is a *c*. 8‰ change in δ^2^H for a given 1‰ change in δ^18^O (Craig, 1961; Rozanski *et al*., 1993). Movement of the two isotopologues H_2_^18^O and ^2^HHO within the leaf may also vary, for example due to different diffusivities in water (Cuntz *et al*., 2007), in air (Barbour *et al*., 2017), and potentially across membranes (Mamonov *et al*., 2007) and there can be different extents of exchange with organic molecules (Yakir, 1992; Chen *et al*., 2020). Here, we aimed to assess whether hydrogen and oxygen stable isotopes in leaf water respond differently to the environment, to better understand whether δ^2^H and δ^18^O in organic matter proxies capture environmental signals differently.

To do this, we compiled datasets that provided measurements under natural conditions of both δ^2^H and δ^18^O in leaf water, xylem water and atmospheric vapour, along with concurrent measurements of air temperature and relative humidity. Table 1 provides a summary of data sources. Within each dataset, we averaged individual observations, such that each row of data in the compiled dataset represents a mean value for a given species by site by time combination. In total, the dataset contained 546 such rows. The geographic range of the combined dataset covered more than 100° of latitude and more than 3000 m of elevation (Table 1). We limited the dataset to daytime observations, as it is primarily during photosynthesis that leaf water signals are incorporated into organic compounds. This also helped to avoid issues of nonsteady state leaf water enrichment at night (Cernusak *et al*., 2002, 2005; Seibt *et al*., 2006). We note that it has recently been shown that extraction of stem xylem water for isotopic analysis can be accompanied by an offset in δ^2^H from the water that is likely to have been taken up by the roots (Zhao *et al*., 2016; Chen *et al*., 2020; Barbeta *et al*., 2022). We did not attempt to apply a correction for this offset as we lacked a basis on which to make the correction that could be applied across the compiled dataset.

**Table 1:** Datasets and associated site information for the data compilation presented in this paper.

Dataset	Site	Latitude	Longitude	Elevation (m)	MAP (mm)	MAT (°C)	Vegetation type	References
Western_USA_Roden	Cascade_Heads	45.03	−123.91	14	2410	10.7	Forest	(Roden & Ehleringer, 2000a,b)
	Bill_Williams_River	34.26	−114.03	150	97	23.8	Woodland	
	Weber_River	41.13	−111.90	1450	510	10.6	Woodland	
	Red_Butte_Canyon	40.78	−111.80	1790	700	10.1	Woodland	
	Big_Cottonwood	40.62	−111.73	1987	840	9.4	Woodland	
Washington_USA_Lai	Wind_River	45.82	−121.95	371	2467	8.7	Forest	(Lai & Ehleringer, 2011)
Utah_USA_Flanagan	Coral_Pink	37.04	−112.72	1855	380	10.5	Woodland	(Flanagan *et al*., 1993)
Tibetan_Plateau_Yu	Lhasa	29.65	91.03	3658	460	8.4	Grassland	W. Yu, unpublished
Qld_Aus_Munksgaard	Cairns	−16.79	145.69	30	2000	25.0	Forest/Woodland	(Munksgaard *et al*., 2017)
	Tinaroo	−17.17	145.54	680	1400	22.0	Forest/Woodland	
	Herberton	−17.34	145.42	918	1150	19.0	Woodland	
	Wild_River	−17.65	145.28	860	950	21.0	Woodland	
	Mount_Garnet	−17.67	145.10	660	800	24.0	Woodland	
NW_China_Zhao	Pailugou_2900	38.54	100.30	2900	369.2	0.7	Forest	(Zhao *et al*., 2014)
	Pailugou_2700	38.55	100.29	2780	369.2	0.7	Forest	
	Riparian	42.02	101.23	930	34.9	8.9	Woodland	
	Gobi	42.27	101.12	906	34.9	8.9	Woodland	
NT_Aus_Cernusak	Alice_Springs	−23.70	133.83	598	276	21.0	Woodland	(Kahmen *et al*., 2013a; Cernusak *et al*., 2016)
	Tennant_Creek	−19.65	134.16	365	454	25.9	Woodland	
	Elliot	−17.50	133.51	234	604	26.8	Woodland	
	Katherine	−14.48	132.36	143	1140	27.2	Woodland	
	Darwin	−12.44	130.88	33	1736	27.6	Woodland	
NSW_Aus_Twining	Tumbarumba	−35.66	148.15	1249	1900	9.6	Forest	(Twining *et al*., 2006)
Hawaii_USA_Kahmen	MLM_1	19.69	155.20	683	5676	18.4	Forest	(Kahmen *et al*., 2011)
	MLM_3	19.66	155.47	2061	2000	11.3	Forest/Woodland	
	MLM_4	19.59	155.45	2465	1500	9.9	Forest/Woodland	
	MLM_5	19.83	155.82	694	500	20.0	Forest/Woodland	
Greenland_Bush	Kangerlussuaq	67.02	−50.70	50	140	−5.7	Grassland	(Bush *et al*., 2017)
Germany_Hirl	Grünschwaige	48.40	11.75	448	743	9.3	Grassland	(Hirl *et al*., 2019)
Germany_Bögelein	Palatinate	49.28	7.81	550	1067	7.9	Forest	(Bögelein *et al*., 2017)
France_Wingate	LeBray	44.71	−0.77	62	900	13.0	Forest	L. Wingate & J. Ogée, unpublished
France_Barbeta	Ciron	44.38	−0.31	60	813	12.9	Forest	A. Barbeta, unpublished
Canada_Flanagan	Lethbridge	49.69	−112.83	910	380	5.8	Grassland	(Flanagan *et al*., 1991a)

## δ^2^H and δ^18^O of leaf water, xylem water and vapour

The Craig–Gordon equation (Craig & Gordon, 1965) forms the basic building block for models of leaf water isotopic composition and provides a convenient entry point for examining the environmental drivers of leaf water δ^2^H and δ^18^O. The Craig–Gordon equation can be approximated as:

(Eqn 1) $$\delta_{e}\;\approx \;{\delta_{s}+\varepsilon }^{+}+\varepsilon_{k}+\left(\delta_{v}‐\delta_{s}‐\varepsilon_{k}\right)h$$

(δ_e_, predicted δ^2^H or δ^18^O at the evaporative sites within leaves; δ_s_, δ^2^H or δ^18^O of source water, which we equated in our dataset to xylem water; ε^+^, equilibrium fractionation between liquid and vapour; ε_k_, kinetic fractionation during diffusion through the stomata and boundary layer; δ_v_, δ^2^H or δ^18^O of atmospheric vapour and *h*, *w*_a_/*w*_i_, the water vapour mole fraction in the air outside the leaf boundary layer divided by that at the evaporative sites inside the leaf substomatal cavity). The *w*_i_ is typically assumed to be saturated at leaf temperature, although recent evidence has suggested that it may be less than saturated at times (Cernusak *et al*., 2018, 2019; Buckley & Sack, 2019; Holloway‐Phillips *et al*., 2019). If *w*_i_ is saturated and leaf temperature is equal to air temperature, then *w*_a_/*w*_i_ is equal to the relative humidity of the air surrounding the leaf. Eqn 1 is an approximation of a more precise form of the Craig–Gordon equation (Farquhar *et al*., 2007); however, it is very useful in that it shows intuitively what the different drivers of δ_e_ are expected to be. Therefore, we used the more precise version of the equation for calculations and analyses, but used the approximate version here to guide our discussion. A summary of formulae for calculating the equilibrium and kinetic fractionation factors for δ^18^O and δ^2^H and the more precise version of the Craig–Gordon equation can be found in Cernusak *et al*. (2016) and in the Supporting Information Dataset S1.

Eqn 1 assumes isotopic steady state, in which the water leaving the leaf through transpiration has the same isotopic composition as that entering the leaf from the xylem. Furthermore, it makes a prediction for the evaporative sites, while the unit of measure in our dataset is bulk leaf water (δ_l_), the total sum of water extracted from the leaf. Bulk leaf water can be expected to be somewhat less enriched than the evaporative sites, due to the influx of unenriched xylem water in the veins (Roden & Ehleringer, 1999; Farquhar & Gan, 2003; Farquhar *et al*., 2007; Holloway‐Phillips *et al*., 2016). Whereas the mechanisms in the Craig–Gordon equation are identical for δ^2^H and δ^18^O, the relative magnitudes of the equilibrium and kinetic fractionation factors differ. For δ^2^H, the ε^+^ is relatively large and ε_k_ relatively small, whereas the converse is true for δ^18^O (Merlivat, 1978; Horita & Wesolowski, 1994; Cernusak *et al*., 2016). The ratio ε^+^ : ε_k_ is *c*. 3 : 1 for δ^2^H and 1 : 3 for δ^18^O (Dataset S1).

**Fig. 1:**
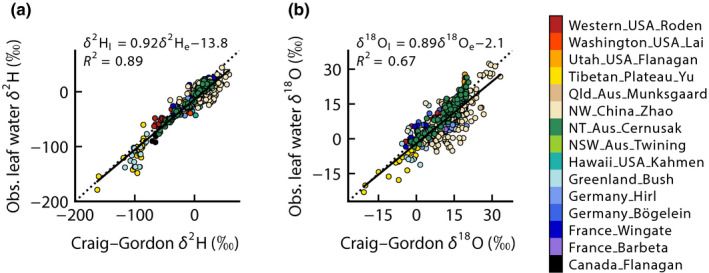
Observed leaf water isotopic composition for δ^2^H (a) and δ^18^O (b) plotted against values predicted by the Craig–Gordon equation. Symbols with different colours refer to the different datasets that have been compiled for this paper. The dotted lines show one‐to‐one lines and solid lines show least‐squares linear regressions with fitted coefficients shown in the panels along with the coefficient of determination, *R* ^2^.

We plotted the Craig–Gordon predicted leaf water isotopic compositions against observations for our dataset, to determine whether the Craig–Gordon equation could provide a reasonable framework for guiding analyses of different drivers. Fig. 1 shows the observed bulk leaf water δ^2^H and δ^18^O plotted against that predicted by the Craig–Gordon equation, using the measured air temperature, relative humidity, isotopic composition of xylem water and atmospheric vapour. Overall, the Craig–Gordon equation explained 89% of observed variation in leaf water δ^2^H and 67% of observed variation in leaf water δ^18^O. As anticipated, the slopes of the relationships were less than unity, as would be the case if some fraction of bulk leaf water represented unenriched xylem water. The generally good predictive ability of the Craig–Gordon equation for daytime leaf water isotopic composition suggests that it can provide a framework for evaluating whether different environmental drivers predominate for hydrogen vs oxygen.

Some additional sources of unexplained variation in Fig. 1 could include departures from isotopic steady state (Dongmann *et al*., 1974; Farquhar & Cernusak, 2005), variation in the fraction of unenriched water in leaves associated with differences in leaf anatomy and physiology (Holloway‐Phillips *et al*., 2016; Barbour *et al*., 2021) and unaccounted for variation in boundary layer conductance (Buhay *et al*., 1996). The detailed data required to test for each of these possibilities were not available across the compiled dataset. However, we did repeat our analyses with observations limited to the middle of the day (from 11:00 h to 14:00 h), when isotopic steady state is most likely to be achieved (Harwood *et al*., 1998). This yielded very similar results to those shown in Fig. 1. The same was also true for subsequent figures and we therefore present analyses with all daytime observations included.

**Fig. 2:**
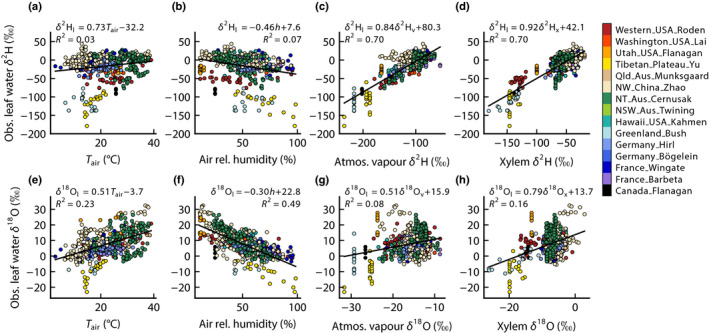
Observed isotopic composition for leaf water δ^2^H (a–d) and δ^18^O (e–h) plotted against the four environmental drivers in the Craig–Gordon equation: air temperature (a, e), air relative humidity (b, f), the corresponding isotopic composition of atmospheric vapour (c, g) and the corresponding isotopic composition of xylem water (d, h). The symbol colours show the different datasets compiled for this paper. Solid lines are least‐squares linear regressions, with fitted coefficients shown in the panels, along with the coefficient of determination, *R* ^2^.

The environmental drivers that are used in the Craig–Gordon equation are air temperature, which impacts ε^+^ (Horita & Wesolowski, 1994); relative humidity, which is assumed equal to *w*_a_/*w*_i_ if leaf temperature has not deviated from air temperature and *w*_i_ is saturated; isotopic composition of source water entering the leaf, assumed equal to the measured xylem water in our analyses; and the isotopic composition of atmospheric water vapour. Fig. 2 shows the observed leaf water δ^2^H and δ^18^O plotted against each of these four environmental drivers. For δ^2^H_l_, xylem water δ^2^H and atmospheric vapour δ^2^H were much more strongly correlated with it than air temperature or relative humidity. For δ^18^O_l_, conversely, air relative humidity was much more strongly correlated than any of the other drivers. For δ^2^H_l_, either xylem water or atmospheric vapour δ^2^H explained more than two‐thirds of its variation, whereas for δ^18^O_l_ the air relative humidity explained about half of its variation.

**Fig. 3:**
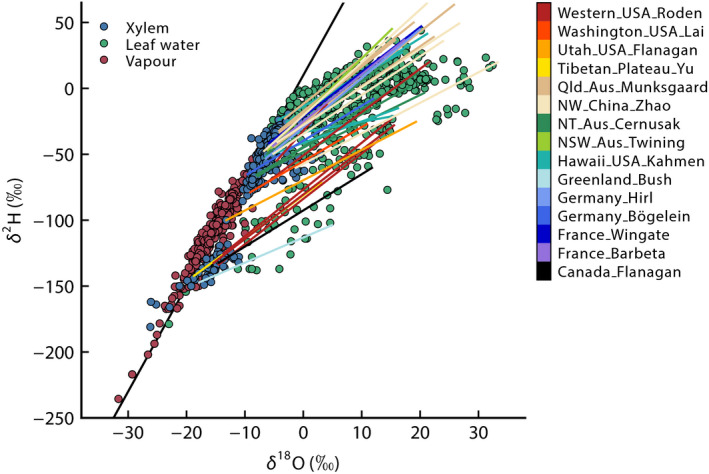
The isotopic composition of xylem water, leaf water and atmospheric vapour plotted in δ^2^H–δ^18^O dual‐isotope space. The black line shows the meteoric water line, defined as δ^2^H = 8 × δ^18^O + 10. The coloured lines show the evaporation lines for leaf water, in which the intercept with the meteoric water line is the mean for each site and the slope is calculated as (δ^2^H_l_ − δ^2^H_x_)/(δ^18^O_l_ − δ^18^O_x_) using the mean quantities for each site, where subscript ‘l’ refers to leaf water and ‘x’ to xylem water. The colours of the lines refer to the individual datasets compiled for this paper. The range of δ^18^O_l_ observed for each site defines the length of the coloured lines.

The reason that xylem water is a much stronger driver of leaf water for δ^2^H than for δ^18^O is because the range of variation in meteoric water isotopic composition compared with that in leaf water evaporative enrichment is larger for δ^2^H than for δ^18^O. This can be seen through inspection of Fig. 3, which shows the evaporation lines for leaf water for each site in the dataset and their extrapolation to the meteoric water line. The range in the *y*‐axis over which the evaporation lines intersect the meteoric water line for δ^2^H is *c*. 120‰ and the corresponding range on the *x*‐axis for δ^18^O is *c*. 15‰, for a ratio of *c*. 8 : 1, consistent with the slope of the meteoric water line. Conversely, the range for leaf water isotopic composition beginning at the meteoric water line and moving right along the evaporation lines is *c*. 100‰ on the *y*‐axis for δ^2^H and *c*. 30‰ on the *x*‐axis for δ^18^O, for a ratio of *c*. 3 : 1. Therefore the point at which the evaporation line intersects the meteoric water line can exert a much stronger influence on leaf water for δ^2^H than for δ^18^O, because its range is relatively large compared with the range over which evaporation can enrich the leaf water above source water. Another way to understand this conceptually is to consider that the slope of the meteoric water line, defining source water variation in δ^2^H–δ^18^O space, corresponds approximately to the ratio of the equilibrium fractionations for δ^2^H and δ^18^O (mean = 8.6 in our dataset). Conversely, the slopes of the evaporation lines corresponded approximately to the ratio of the sum of equilibrium and kinetic fractionations (mean = 2.9 in our dataset).

This difference between leaf water dynamics for δ^2^H and δ^18^O, driven by source water isotopic composition, is important for interpreting organic material signals. For example, leaf water proxies based on δ^2^H, such as the δ^2^H of *n*‐alkanes derived from leaf waxes, if sampled across a large geographic range, could be expected to be strongly influenced by a widely varying δ^2^H of source water (Liu & Yang, 2008; Sachse *et al*., 2012; Ladd *et al*., 2021). Conversely, only if there is little variation in source water δ^2^H, will the variation in *n*‐alkane δ^2^H of leaf waxes reliably record the extent of leaf water evaporative enrichment (Kahmen *et al*., 2013b). For an organic matter proxy such as cellulose δ^18^O, we would expect the geographic variation in source water isotopic composition to have less influence compared with the dynamics of leaf water enrichment above source water, driven primarily by relative humidity (Barbour & Farquhar, 2000; Kahmen *et al*., 2011). To the extent that such geographic variation can provide a space for time substitution, our results also have implications for interpreting changes through time within a site. For example, δ^2^H of *n*‐alkanes from leaf waxes has been combined with δ^18^O of hemicellulose sugars for reconstructing paleoclimate from sedimentary records (Zech *et al*., 2013; Hepp *et al*., 2021). Our results suggest that δ^2^H of *n*‐alkanes should be better suited to detecting changes in δ^2^H of precipitation and δ^18^O of hemicellulose sugars to detecting changes in relative humidity. We note, however, that the extent of transfer of the leaf water signal to the biomarker will also be important; for example, for cellulose δ^18^O, our analysis of leaf water δ^18^O may be more relevant to leaf cellulose than to stem wood cellulose, as the latter is subject to partial exchange with unenriched xylem water (Roden *et al*., 2000; Kahmen *et al*., 2011; Cheesman & Cernusak, 2017), with the same caveat also applicable for grasses (Helliker & Ehleringer, 2000; Liu *et al*., 2017).

## Isotopic enrichment of leaf water above xylem water

**Fig. 4:**
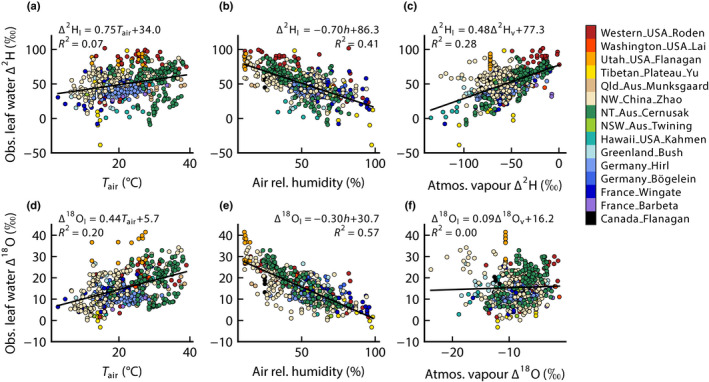
Leaf water isotopic enrichment above xylem water for hydrogen, Δ^2^H (a–c), and for oxygen, Δ^18^O (d–f), plotted against air temperature (a, d), air relative humidity (b, e) and the corresponding isotopic enrichment of atmospheric vapour above xylem water (c, f). Solid lines show least‐squares linear regressions, along with fitted coefficients and the coefficient of determination, *R* ^2^. Symbol colours refer to individual datasets compiled for this paper.

In addition to xylem water δ^2^H having a close correlation with leaf water δ^2^H, atmospheric vapour δ^2^H also had a close correlation. The next question we asked in our analysis was whether the relationship between leaf water and vapour for δ^2^H would still remain stronger than that for δ^18^O when variation in xylem water isotopic composition was removed. To answer this question, we expressed leaf water as enrichment above xylem water (Δ_l_), calculated as Δ_l_ = (δ_l_ − δ_x_)/(1 + δ_x_), where the subscript ‘l’ refers to leaf water and ‘x’ to xylem water. Again, we use the approximate form of the Craig–Gordon equation here for ease of interpretation to guide our analysis, but used the more precise form in our calculations. With leaf water expressed as enrichment above source water, the Craig–Gordon equation becomes (Farquhar *et al*., 1989):

(Eqn 2) $$\Delta_{e}\;\approx \;\varepsilon^{+}+\varepsilon_{k}+\left(\Delta_{v}‐\varepsilon_{k}\right)h$$

(Δ_e_, predicted enrichment at the evaporative sites in leaves and Δ_v_, enrichment of atmospheric vapour relative to source water). Note that this latter term is generally negative; that is, atmospheric vapour is generally depleted in heavier isotopes compared with source water. In our analysis, we calculated Δ_v_ as Δ_v_ = (δ_v_ − δ_x_)/(1 + δ_x_), where δ_v_ is δ^2^H or δ^18^O of atmospheric vapour and δ_x_ is that of xylem water. In Fig. 4, we show the observed bulk leaf water enrichment plotted against the three environmental drivers remaining in Eqn 2.

Fig. 4 shows that the correlation between leaf water and relative humidity for hydrogen has been markedly improved by removing source water variation, with relative humidity now explaining 41% of the variation in Δ^2^H_l_. Therefore, after calculating Δ^2^H_l_ to remove the source water signal, the sensitivity to relative humidity became more apparent. For oxygen, there was also a strengthening of the correlation between leaf water enrichment and relative humidity, with the *R* ^2^ increasing from 0.49 to 0.57. Interestingly, however, the correlation between leaf water enrichment and atmospheric vapour enrichment was still relatively strong for hydrogen, but weak for oxygen, which stands out as a point of difference between Δ^2^H and Δ^18^O in Fig. 4. Stronger relationships with atmospheric vapour for Δ^2^H_l_ than for Δ^18^O_l_ have also been observed previously in some of the individual datasets that have now been compiled for this paper (Cernusak *et al*., 2016; Bögelein *et al*., 2017; Munksgaard *et al*., 2017).

## Role of atmospheric vapour isotopic composition

Can we identify further the underlying cause of the stronger correlation between leaf water and atmospheric vapour for Δ^2^H compared with Δ^18^O? To explore this, we turned again to the Craig–Gordon equation, taking the derivative of Eqn 2 with respect to Δ_v_. This provides a mathematical description of predicted drivers of the change in Δ_l_ for a given change in Δ_v_:

(Eqn 3) $$\frac{d\Delta_{l}}{d\Delta_{v}}=\frac{d\varepsilon^{+}}{d\Delta_{v}}+\frac{d\varepsilon_{k}}{d\Delta_{v}}+h\left(1‐\frac{d\varepsilon_{k}}{d\Delta_{v}}\right)+\left(\Delta_{v}‐\varepsilon_{k}\right)\frac{dh}{d\Delta_{v}}$$

We used our dataset to estimate the terms in Eqn 3 by taking regression slopes for the derivative terms and mean values for *h* and (Δ_v_ − ε_k_). These estimates are shown in Table 2 for Δ^2^H and Δ^18^O. From Table 2, it can be seen that the first two terms on the right side of Eqn 3 are small in magnitude for both Δ^2^H and Δ^18^O and unlikely to have a strong influence on dΔ_l_/dΔ_v_ for either. Because dε_k_/dΔ_v_ is small, it means that the third term on the right side will approach the value of *h*, which is larger by comparison, having a mean value in our dataset of 0.5, or an air relative humidity of *c*. 50%. The largest term in Eqn 3 by far for both Δ^2^H and Δ^18^O is (Δ_v_ − ε_k_), having mean values of −79‰ and −35‰ for Δ^2^H and Δ^18^O, respectively. This is then multiplied by a much smaller term, d*h*/dΔ_v_. Importantly, (Δ_v_ − ε_k_) is negative, setting up the possibility that the interplay between the third and fourth terms on the right side of the equation could be important, with the third term, *h*(1 − dε_k_/dΔ_v_), being positive and the fourth term, (Δ_v_ − ε_k_)d*h*/dΔ_v_, potentially counteracting it with a negative value.

**Table 2:** Values for the terms in Eqn 3 calculated from the combined dataset.

	$\frac{{d\varepsilon }^{+}}{{d\Delta }_{v}}$	$\frac{{d\varepsilon }_{k}}{{d\Delta }_{v}}$	h	$\left(\Delta_{v}‐\varepsilon_{k}\right)$	$\frac{dh}{{d\Delta }_{v}}$	$h\left(1‐\frac{{d\varepsilon }_{k}}{{d\Delta }_{v}}\right)$	$\left(\Delta_{v}‐\varepsilon_{k}\right)\frac{dh}{{d\Delta }_{v}}$	Sum of shaded columns
Δ^2^H	−0.09	−0.01	0.51	−79.2	−0.0004	0.51	0.03	0.45
Δ^18^O	0.01	−0.02	0.51	−35.2	0.0102	0.52	−0.36	0.15

Derivative terms (d*y*/d*x*) were calculated as the slope of a linear regression of the two parameters *y* and *x*, whereas nonderivative terms were calculated as the mean of the given parameter. According to Eqn 3, the dependence of leaf water isotopic enrichment on the atmospheric vapour isotopic composition, dΔ_L_/dΔ_v_, is equal to the sum of the shaded columns, which is shown in the final column. As can be seen, the primary difference for Δ^2^H compared with Δ^18^O results from the term d*h*/dΔ_v_; that is, a correlation between atmospheric humidity and the isotopic composition of atmospheric vapour, which is much stronger for Δ^18^O than for Δ^2^H.

The interaction between the third and fourth terms in Eqn 3 does indeed appear to be pivotal in explaining why leaf water correlates more strongly with atmospheric vapour for Δ^2^H than for Δ^18^O. For Δ^2^H, the linear regression between *h* and Δ^2^H_v_ was not significant (*P* = 0.32, *n* = 546) and had a slope of −0.0004. This gives a value for the fourth term in Eqn 3 for Δ^2^H of 0.03, which therefore adds slightly to the positive value of the third term, again having a value of *c*. 0.5. Conversely, for Δ^18^O, the regression between *h* and Δ^18^O_v_ was significant (*P* < 0.001, *n* = 546) and had a positive slope of 0.0102. Because this slope was positive, the fourth term on the right side of Eqn 3 for Δ^18^O takes on an overall negative value of −0.36. Therefore, for Δ^18^O, the fourth term on the right side of Eqn 3 largely cancels the influence of the third term and, as a result, there is little change in Δ^18^O_l_ for a given change in Δ^18^O_v_. This manifests in our dataset as a weakened correlation between leaf water and atmospheric vapour for Δ^18^O as seen in Fig. 4f whereas, for Δ^2^H, there is a stronger correlation between leaf water and atmospheric vapour, as seen in Fig. 4c.

The analysis above shows that there is a positive correlation between relative humidity and the Δ^18^O of atmospheric vapour in our dataset, whereas such a correlation does not exist between relative humidity and the Δ^2^H of atmospheric vapour. Through application of Eqn 3, we showed that this difference partly explains why leaf water δ^2^H more strongly correlates with atmospheric vapour δ^2^H than is the case for δ^18^O. Another way to approach the underlying issue of this apparent difference in behaviour of atmospheric vapour for the two isotopologues with respect to relative humidity is to calculate the deuterium excess or the departure from an expectation of the relationship between δ^2^H and δ^18^O based on the meteoric water line. The global meteoric water line is described by δ^2^H = 8 × δ^18^O + 10 (Craig, 1961). We therefore calculated the deuterium excess of water vapour, *d*_v_, as *d*_v_ = δ^2^H_v_ – 8 × δ^18^O_v_ (Dansgaard, 1964). We also made the same calculation for atmospheric vapour composition with respect to xylem water, Δ_dv_ = Δ^2^H_v_ – 8 × Δ^18^O_v_. We then tested for correlations between these parameters and the air relative humidity in our dataset. Both showed significant negative correlations with relative humidity, with the relationship stronger for Δ_dv_ (*R* ^2^ = 0.17, *P* < 0.001, *n* = 546) than for *d*_v_ (*R* ^2^ = 0.08, *P* < 0.001, *n* = 546).

A relationship between the deuterium excess of atmospheric vapour and relative humidity has also been observed on diurnal timescales at six sites in the northern hemisphere (Welp *et al*., 2012) and at a tropical site in Cairns, Australia (Munksgaard *et al*., 2020). This pronounced, general pattern is thought to be driven by the diurnal pattern of plant transpiration and the contribution of transpired water to atmospheric vapour and by entrainment of the free atmosphere into the planetary boundary layer with increased convective mixing during the day. The result is a general midday decrease in the δ^18^O of atmospheric vapour, but little to no change in δ^2^H. This leads to the diurnal variability of *d*_v_, which is anticorrelated with the diurnal pattern of relative humidity (Welp *et al*., 2012; Munksgaard *et al*., 2020). A shorter time series of 3 d at the Wind River Experimental Forest (Washington, USA) showed a similar pattern (Lai & Ehleringer, 2011). The strength of this pattern suggests that such diurnal variation could be driving the overall relationship between *d*_v_ and relative humidity in our dataset. Welp *et al*. (2012) also observed negative correlations between day‐to‐day variation in *d*_v_ and relative humidity throughout the summer months at sites located near large bodies of water, with such patterns also previously reported for sites in marine‐type settings (Uemura *et al*., 2008; Gat *et al*., 2011). Such dynamics related to marine air sources may also have been relevant at some sites within our dataset. When we restricted our analysis from daytime observations to only midday observations (between 11:00 h and 13:00 h) to minimise diurnality, we observed a weak, but still significant, relationship between *d*_v_ and relative humidity (*R* ^2^ = 0.02, *P* < 0.05, *n* = 200), showing the importance of diurnal effects. What is clear overall is that covariation between Δ^18^O_v_ and relative humidity, but not Δ^2^H_v_ and relative humidity, plays an important role in modulating leaf water isotope dynamics, leading to the result highlighted in our dataset of Δ^2^H of leaf water showing stronger correlation with Δ^2^H of atmospheric vapour than is the case for Δ^18^O.

## Are two isotopes better than one?

Some organic matter proxies, such as plant cellulose, allow both δ^18^O and δ^2^H to be measured. In such cases, it is possible to estimate leaf water isotopic composition for both δ^2^H and δ^18^O and therefore to reconstruct *d*_l_, the deuterium excess for leaf water. As seen in Fig. 3, the slopes of the evaporation lines tend to be uniform and independent of the source water and the position along the evaporation line is determined mainly by relative humidity. Therefore, *d*_l_ is expected to show a relationship with relative humidity that is largely independent of source water isotope composition. For this reason, it has been suggested that such a dual‐isotope approach could provide a stronger basis for reconstructing relative humidity than either isotope alone when the source water isotopic composition is not known (Zech *et al*., 2013; Voelker *et al*., 2014). Our dataset gave us an opportunity to test this idea across a diverse range of sites and conditions.

**Fig. 5:**
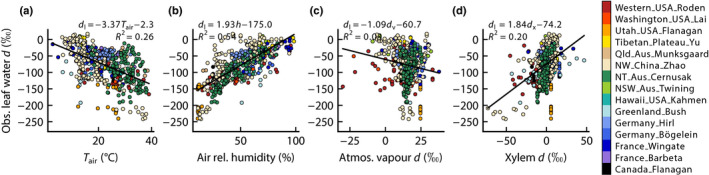
Deuterium excess in leaf water plotted against (a) air temperature, (b) air relative humidity, (c) the deuterium excess in atmospheric water vapour and (d) the deuterium excess in xylem water. The deuterium excess, *d*, was calculated as *d* = δ^2^H – 8 × δ^18^O. Solid lines show least‐squares linear regressions, along with fitted coefficients and the coefficient of determination, *R* ^2^. Symbol colours refer to individual datasets compiled for this paper.

Fig. 5 shows *d*_l_ for the dataset, calculated as *d*_l_ = δ^2^H_l_ – 8 × δ^18^O_l_, plotted against potential drivers, including relative humidity. Because *d*_l_ was calculated from δ^18^O_l_ and δ^2^H_l_, we can compare the relationships with relative humidity among the three. The *d*_l_ does indeed have the strongest relationship; however, it is not very much stronger than that for δ^18^O_l_. The *R* ^2^ for *d*_l_ vs relative humidity is 0.54 (Fig. 5b), whereas that for δ^18^O_l_ is 0.49 (Fig. 2f), and that for δ^2^H_l_ is 0.07 (Fig. 2b). Therefore, surprisingly, leaf water δ^18^O on its own would be nearly as good a predictor of air relative humidity as *d*_l_ calculated from both the δ^18^O and δ^2^H of leaf water. The explanation for this is the relatively constrained variation in δ^18^O of xylem water, especially in comparison with δ^2^H (Fig. 3) and the relative insensitivity of Δ^18^O_l_ to atmospheric vapour Δ^18^O_v_, as discussed above. Given these considerations, we suggest that if applying a dual‐isotope approach in this way, one should weigh up carefully the uncertainty associated with estimating leaf water δ^2^H from cellulose δ^2^H, given the relative complexity in the signal transfer pathway from leaf water to cellulose for δ^2^H (Cormier *et al*., 2018; Lehmann *et al*., 2021). The cost in uncertainty associated with this may not be worth the relatively modest improvement in strength of the correlation between *d*_l_ and relative humidity compared with that for δ^18^O alone.

## Conclusions

We compiled a dataset for δ^2^H and δ^18^O of leaf water, xylem water, atmospheric vapour, air temperature and relative humidity from a diverse range of sites and used the dataset to test for differences in how leaf water δ^2^H and δ^18^O reflect environmental drivers. We conducted the analysis in the context of asking which drivers could be best reconstructed from leaf water proxies based on organic material. Xylem water δ^2^H was a much stronger driver of variation in leaf water δ^2^H than was the case for xylem water δ^18^O as a driver of variation in leaf water δ^18^O. Conversely, relative humidity showed a considerably stronger relationship with leaf water δ^18^O than it did with leaf water δ^2^H. This pattern persisted when we removed xylem water isotopic variation from the leaf water signal by expressing the leaf water isotopic composition as an enrichment above xylem water. We identified the underlying reason for this pattern as a correlation between relative humidity and the δ^18^O of atmospheric vapour. Such a correlation has also been observed in time series of vapour isotopic composition measurements, and manifests most clearly as an anticorrelation between the deuterium excess of atmospheric vapour and relative humidity on diurnal timescales. While we did not have sufficient resolution of sampling within sites to tease this apart in our dataset, we suspect that this diurnal pattern is likely to underlie the correlation between relative humidity and atmospheric vapour Δ^18^O_v_ that we observed. We conclude that leaf water δ^2^H and δ^18^O do indeed reflect the balance of potential environmental drivers differently: leaf water δ^2^H reflects more strongly xylem water δ^2^H and atmospheric vapour δ^2^H, whereas leaf water δ^18^O reflects more strongly air relative humidity.

## Author contributions

LAC and MC initiated the review and conducted initial analyses. All authors contributed to further development of ideas and writing of the manuscript.

## Supporting information


**Dataset S1** An Excel file containing the compiled dataset that was analysed for this paper.
Please note: Wiley Blackwell are not responsible for the content or functionality of any Supporting Information supplied by the authors. Any queries (other than missing material) should be directed to the *New Phytologist* Central Office.

## Data Availability

All data used in the analyses presented in this paper are available in Supporting Information Dataset S1.
